# Quantitative Characterization of the Filiform Mechanosensory Hair Array on the Cricket Cercus

**DOI:** 10.1371/journal.pone.0027873

**Published:** 2011-11-21

**Authors:** John P. Miller, Susan Krueger, Jeffrey J. Heys, Tomas Gedeon

**Affiliations:** 1 Center for Computational Biology, Montana State University, Bozeman, Montana, United States of America; 2 Chemical and Biological Engineering, Montana State University, Bozeman, Montana, United States of America; 3 Department of Mathematics, Montana State University, Bozeman, Montana, United States of America; Max-Planck Institute of Neurobiology, Germany

## Abstract

**Background:**

Crickets and other orthopteran insects sense air currents with a pair of abdominal appendages resembling antennae, called cerci. Each cercus in the common house cricket *Acheta domesticus* is approximately 1 cm long, and is covered with 500 to 750 filiform mechanosensory hairs. The distribution of the hairs on the cerci, as well as the global patterns of their movement vectors, have been characterized semi-quantitatively in studies over the last 40 years, and have been shown to be very stereotypical across different animals in this species. Although the cercal sensory system has been the focus of many studies in the areas of neuroethology, development, biomechanics, sensory function and neural coding, there has not yet been a quantitative study of the functional morphology of the receptor array of this important model system.

**Methodology/Principal Findings:**

We present a quantitative characterization of the structural characteristics and functional morphology of the cercal filiform hair array. We demonstrate that the excitatory direction along each hair's movement plane can be identified by features of its socket that are visible at the light-microscopic level, and that the length of the hair associated with each socket can also be estimated accurately from a structural parameter of the socket. We characterize the length and directionality of all hairs on the basal half of a sample of three cerci, and present statistical analyses of the distributions.

**Conclusions/Significance:**

The inter-animal variation of several global organizational features is low, consistent with constraints imposed by functional effectiveness and/or developmental processes. Contrary to previous reports, however, we show that the filiform hairs are not re-identifiable in the strict sense.

## Introduction

An implicit hypothesis underlying recent work in neurophysiology, neuroethology and psychophysics is that sensory systems have evolved, through natural selection, toward optimal functional performance. The functional parameters that are considered for assessing optimality are typically assumed to include stimulus threshold, dynamic range, sensitivity and signal-to-noise characteristics, encoding efficiency, and energetic efficiency. These parameters are, in turn, dependent on the functional packing density and the degree of overlap of the receptive fields of the constituent receptor cells. Many factors that are expected to constrain the processes of optimization have been identified, ranging from limits imposed by chemistry and physics during the transduction process to limits imposed by the process of natural selection, which operates by improvising on “legacy structures”. Ultimately, all operative constraints are expressed during the animal's development, and these developmental processes may also impose additional and, perhaps, overriding constraints on the structural characteristics of a sensory apparatus. Here we present the results of an anatomical study of a simple sensory receptor array, motivated by the ultimate goal of interpreting the details of that structure within the contexts of functional optimality and the constraints imposed on that structure by developmental mechanisms.

The system we studied is the cercal sensory system of the cricket *Acheta domesticus*. This mechanosensory system functions as a low-frequency, near field extension of the animal's auditory system, and mediates the detection, localization and identification of air current signals generated by predators, mates and competitors. The cercal system is crucial for the cricket's survival: on the basis of the information captured by this sensory system, the animal must make decisions rapidly and reliably (*i.e.*, discriminate between different possible signal sources with few false classifications). A general motivating hypothesis for our studies is that the biomechanical and neurophysiological characteristics of the receptor organs for this system have become optimized for the sensory processing operations they mediate, and that the structure of these organs should reflect the extent of this evolutionary optimization. In this paper, we present the results of a quantitative analysis of the structure of the sensory apparatus for the cercal system.

**Figure 1:**
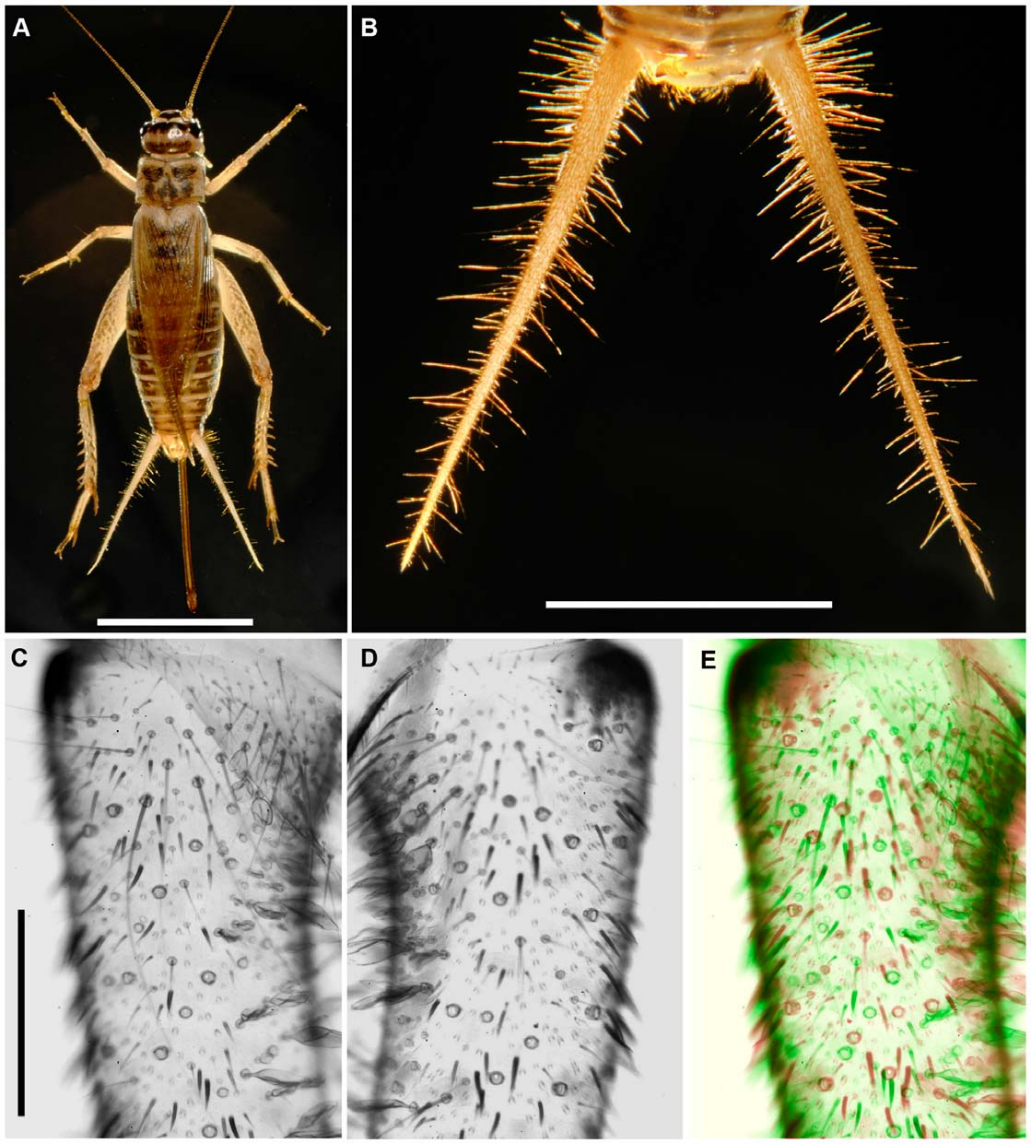
The cerci of adult female *Acheta domesticus* crickets. A. An adult female cricket, with the cerci visible on the posterior of the abdomen on either side of the ovipositor. B. Higher magnification of the cricket's cerci, with ovipositor removed, showing the filiform mechanosensory hairs. C,D. Higher magnification images of the regions near the bases of both (left and right) cerci from a single animal, from which many of the filiform hairs have been plucked from the sockets. E. The image of the right cercus from panel D has been left-right inverted, colored red, and superimposed on a green-hued image of the same segment from right cercus from panel C, to illustrate the variation in the relative location of filiform hair sockets. Scale bars: A: 1 cm, B: 5 mm, C–E *(left, panel* ***c*** *)* ***:*** 500 microns.

The sensory apparatus for the cercal system consists of a pair of antenna-like *cerci* at the rear of the cricket's abdomen (Figure 1). In the adult cricket, each cercus is approximately 1 cm in length, and each is covered with between 500 and 750 filiform mechanosensory hairs ranging in length from 50 microns to almost 2 mm [1]. Each filiform hair is innervated by a single spike-generating sensory receptor neuron [2]. These filiform hairs are extremely sensitive to air currents in the animals' immediate environment, and the deflection of a hair by air currents changes the spiking activity of the associated receptor neuron at the base of the hair. All of the information that the cercal system extracts from air currents in the environment is derived from the combined spiking activity patterns of the array of approximately 1000–1500 hair receptors on both cerci. The functional characteristics of this mechanoreceptor array are determined by the bio-mechanical structural characteristics of the filiform hairs and by the distribution of hairs on the cerci. These are the aspects which have been shown to display low inter-animal variance, and the current consensus is that filiform hairs are essentially re-identifiable: *i.e.*, that a filiform hair with a particular set of structural and biomechanical properties can be found at the same relative location on every normal cercus [1], [3], [4]. It was precisely that aspect of the filiform sensory array that were measured and analyzed in this study, and we come to a different conclusion: there is considerable inter-animal variance, and individual filiform mechanoreceptors are *not* strictly re-identifiable.

The specific aspects of the cercal filiform hair array that we characterized were a) the lengths of the hairs, b) the specific pattern of the locations of the hairs on the cerci, and c) the global pattern of the optimal movement directions of the hairs. The functional significance of filiform hair length has been the subject of many previous studies: a hair's length is a primary determinant of it's mechanical sensitivity to air currents, and the overall sensitivity and frequency bandwidth of the mechanosensory array depends upon the distribution of hair lengths and packing densities [5]–[21]. The sensitivity of the sensor array to the direction of air currents emerges from a different aspect of the hair biomechanics, however. Each filiform hair is constrained to move back and forth through a single plane by a cuticular hinge at the base of the hair. Movement of a hair in one direction along that plane leads to excitation of the associated mechanosensory receptor neuron, and movement in the opposite direction inhibits the neuron [22]. Different hairs have different vectors of motion, and all of those movement vectors are stimulated by air movements in the horizontal plane. The ensemble of 500 hairs on each cercus have movement vectors that cover all possible directions in the horizontal plane, though that distribution is non-uniform across directions [1]–[4], [23]–[25]. Note that even though each hair is constrained to a single movement plane, every air current in the horizontal plane will move every hair to some extent: each hair will experience a force that is proportional to the cosine of the angle between its optimal movement vector and the air current vector. The differential directional selectivity of the hairs in the cercal array is extremely important from a functional standpoint: air currents from different directions elicit different patterns of activation across the whole ensemble of the cercal hairs, which in turn enables the cricket to discriminate different stimulus directions.

The goal of the study presented here was to determine the lengths, locations and excitatory movement vectors of all filiform hairs on equivalent segments of cerci from three different adult female crickets, with adequate accuracy and precision to test the hypothesis of inter-animal re-identifiability of the receptors, and to support quantitative tests of other hypotheses emerging from biomechanical [8], [17], [21] and developmental [3], [26] studies. Our protocols for measuring this distribution and directional movement vectors of filiform hairs followed the general approaches used earlier [1]. However, in earlier studies, researchers had not been able to determine the excitatory directional selectivity of a filiform hair through direct observation of the structure of the hair's socket at the light microscopic level. Although it has been possible to determine the direction of motion of large samples of individual hairs by direct observation of their movements, observation of a hair's movement plane alone does not enable discrimination between the excitatory direction *vs.* the inhibitory direction along that plane. Earlier reports of the excitatory direction of a hair have depended upon the simultaneous recording of the mechanosensory receptor neuron during the deflection of the hair [4], [9], [25] or upon electron microscopic imaging of the base of a hair [22], [27], [28], both of which preclude a systematic analysis of a complete set of hairs on the base of a cercus. We demonstrate that aspects of the structure of the socket known to correspond to the excitatory direction of movement can be observed at the light microscopic level, enabling a comprehensive characterization of the directionality of the receptor array. Furthermore, other aspects of the structure of a hair socket, that are observable at the light microscopic level, correlate very highly with the length of the hair associated with that socket. Thus, we were also able to estimate the length of the filiform hair associated with each socket we characterized.

## Materials and Methods

### Dissection and preparation of cricket cerci

All experiments were performed on adult female *Acheta domesticus* crickets, obtained from Bassett's Cricket Ranch (Visalia, CA). For all experiments, we took considerable care to select crickets that were essentially identical in length and appearance, and had fully intact cerci of identical length with no visible damage. This precluded, as much as possible, the inclusion of animals that had experienced damage to a cercus during some earlier instar: such animals have one cercus that is smaller than another, or two cerci that are either non-uniform in shape or smaller than one cm in total length. Each cricket specimen was selected within four hours following the final molt, and anesthetized by chilling until it ceased to show movement when touched (approximately 5 minutes). The head, legs, wings and ovipositor were removed. Each of the two cerci, along with the small ring of cuticle surrounding the base of each cercus, were cut away from the rear of the abdomen. For each cercus, the tip of a pair of very fine dissecting scissors was inserted into the open (basal) end, and the cercus was cut out along its medial aspect to at least one half of its total length. The distal (un-cut) segment of the cercus was removed and discarded. The remaining basal segment was placed in 1N KOH for 12 hours at 65°C. This treatment dissolved all tissue except the cuticle, and also softened the cuticle to enable its subsequent flattening onto a microscope slide. The tissue was then transferred into 70% EtOH at room temperature. The next stages of the preparation depended upon the specific experimental protocol to be used.

### Preparation of microscope slides with isolated filiform hairs

In order to measure the lengths and base diameters of a large sample of filiform hairs, it was necessary to remove the hairs from the cerci and mount them for microscopic imaging. To do so, cerci that had been processed in KOH, as described above, were dehydrated through series alcohol to 100% EtOH, and then transferred to methyl salicylate for clearing. After at least ten minutes in methyl salicylate, a large sample of filiform hairs spanning the entire range of lengths and orientations on the cercus segment were plucked out using fine (#5) forceps, during observation through a stereo dissecting microscope, and placed into a drop of Canada Balsam on a microscope slide. For a small fraction of these hairs, the sockets were observed to have been removed, intact, along with the hairs. A cover slip was placed over the drop containing the hairs. The filiform hairs on these slides were then observed and imaged using a Leica TCS-SP confocal microscope. The filiform sockets and hairs were highly autofluorescent in the green-to-yellow region, when illuminated with the 488 nm line of an Argon laser. The hair length, hair diameter, and dimensions of the ellipsoid socket base were measured from confocal images using the program *ImageJ* [29]. The measurement of hair diameter required a consistent choice for the position of measurement, because the diameter varies substantially below the point at which the hair passes through the socket aperture. For consistency, the diameter of each hair was measured at a distance up from the base of the hair equal to twice the distance from the base of the hair to the bulge in the hair as it passes through the top aperture of the socket. Beyond this point, the diameter of each hair becomes uniform.

### Preparation of flat-mounted “fillets” of cerci

In order to observe the spacing, diameters and orientations of filiform hair sockets in their natural configuration, we developed protocols for uncurling and flattening the cylindrical segments of cerci which had been processed in KOH and cut along their long axis as described above. Two different types of flattened preparations were used in our studies. For the first type, all filiform hairs on the cercus segment were plucked out using fine forceps, during observation through a stereo dissecting microscope. For the second type, the hairs were not plucked, but rather were cut off at their bases as close to the surface of the cercus as possible using very fine, angled dissecting scissors. The tissue was then dehydrated through a series of increasing alcohol concentrations to 100% EtOH, and then transferred to methyl salicylate for clearing. After at least ten minutes in methyl salicylate, the tissue was transferred into a drop of Canada Balsam on a microscope slide. The cylindrical segment of the cercus was then teased open along the longitudinal cut using fine glass probes, and flattened onto the slide, with the inside surface of the cercus oriented downward toward the slide. A cover slip was placed over the specimen. These sliced-opened and flattened segments of cerci will subsequently be referred to as fillet preparations. The final dimensions of these flattened filets was approximately 2×6 mm.

### Microscopy

The specimens were observed and imaged using a Leica TCS-SP microscope, in either of two imaging modes. As described above, the filiform sockets hairs were highly autofluorescent in the green-to-yellow range, and could be imaged in confocal mode when illuminated with the 488 nm line of an Argon laser. The specimens could also be imaged using standard DIC optics using the transmitted light configuration of the microscope. In this DIC mode, a photodetector below the stage collects the light that is transmitted through the image. Although the scanning laser is used to illuminate the tissue, the resulting image is not passed back through a conjugate pinhole before capture by the photodetector, so the images do not have the optical resolution obtainable with the confocal mode.

### Preparation of photographic montages of entire cercal fillets

In order to obtain accurate measurements of the spacing, diameters and orientations of filiform hair sockets in their natural configuration, we needed images of entire filet preparations at a high enough magnification to resolve the structural details of the sockets. Adequate resolution was obtained using DIC imaging with a 20×0.5NA dry objective on the Leica TCS-SP microscope, recorded at a resolution of 2048×2048. Because the dimensions of each filet preparation were much larger than the field of view using a 20× objective, a photographic montage of each cercal fillet was constructed from up to 30 overlapping images, joined together to form a continuous image using *Adobe Photoshop*.

In order to capture all information specifying the position, excitatory movement vector, and filiform hair length, the positions of three characteristic features were recorded for every socket in the fillet montage, using the program *ImageJ*. Those features were the positions of the endpoints of the long axis of the ellipsoidal socket cavity, and the position of the notch at the end of the small axis of the ellipsoidal socket cavity. Illustrative images are presented in the Results section.

## Results

In *Acheta domesticus*, each cercus is approximately 1 cm long in a normal adult cricket. Figure 1A shows an adult female cricket, with the cerci clearly visible on the posterior of the abdomen on either side of the ovipositor. Figure 1B is a photograph of a cricket's cerci in their normal configuration, illuminated so that the filiform mechanosensory hairs are visible. Figures 1C and 1D are higher magnification images of the regions near the bases of both (left and right) cerci from a single animal. The dark circular forms on the cercal surface are the sockets at the base of filiform mechanosensory hairs. Some of the sockets have filiform hairs attached, though many of the hairs have been plucked out, leaving empty sockets. Note that there is noticeable variation in the relative location of filiform hair sockets on the two cerci: the patterns of sockets in these images are not perfectly mirror symmetric. This variability is shown more clearly in Figure 1E, in which the image of right cercus has been left-right inverted, colored red, and superimposed on a green-hued image of the same segment from right cercus. Also note that a sub-class of freely-articulated trichoid sensilla are also visible in panels C and D of Figure 1: the paddle-shaped clavate hairs, located along the medial face of the cerci near the base.

### The excitatory movement directions of filiform hairs can be determined from light microscopic images of the hair sockets

Previous morphological studies have shown that there are structural features at the base of every filiform hair and its associated socket that are unequivocal indicators of the direction of movement of that hair and the excitatory vector along that direction. All of these morphological studies have been carried out at the E.M. level. As we demonstrate below, these features are clearly visible using a light microscope using objectives as low as 20×. The crucial structural features and their functional correlates are as follows.

#### Ellipsoidal socket septum

The base of every filiform hair terminates with a cuticular plate having two slightly rounded lateral projections called articular pegs, which give the base plate an overall ellipsoidal shape. These articular pegs act as trunnions (or pivots), and fit into two bearings in an ellipsoidal cavity in the septum at the base of the socket. This mechanical arrangement constrains the hair to move in a single plane, perpendicular to the major axis of the ellipsoidal hair base defined by the pegs. These features are shown very clearly in several publications using transmission and scanning EM (Figure 1E of [2], Figure 1C of [22], see also [28], [30]). These studies also show how the shape of the cavity through the septum that surrounds the base of the hair in the socket corresponds precisely to the shape of the hair base.

**Figure 2:**
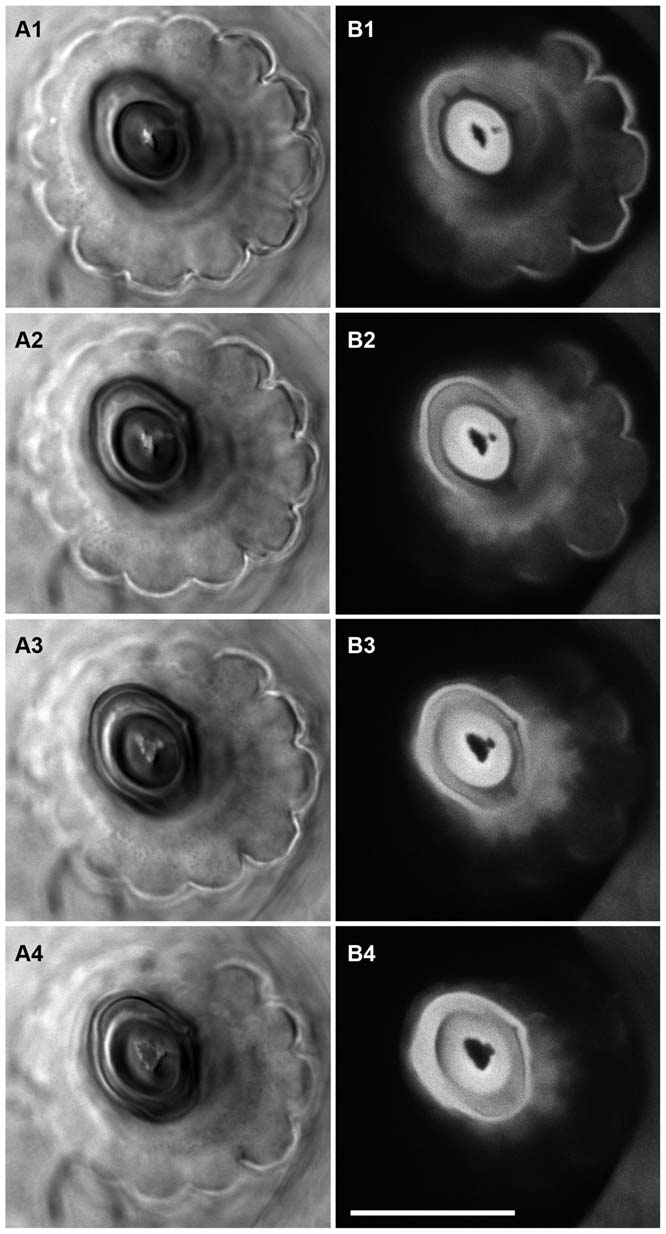
Sequential serial optical sections through the base of a filiform hair socket. The base of the filiform hair is intact in these sections. The sections were taken from directly above the socket, along the long axis of the cut-off hair. Images were taken with a 100X Zeiss Neoufluar oil immersion objective with NA of 1.0. A1–A4. Images on the left were obtained with Koehler illumination in DIC mode. B1–B4. Images on the right were obtained in fluorescence confocal mode. Each horizontal pair was obtained at the same image plane, with the deepest plane nearest the base of the hair in the bottom pair of panels (A4 and B4). Scale bar: 20 microns.

A hair's movement plane can therefore be determined by direct observation of the socket: the major axis of the ellipsoidal septum cavity and hair base is perpendicular to the plane of hair movement. Figure 2 shows a sequential set of serial optical sections through a filiform hair socket in which the base of filiform hair was still intact (the hair was cut off just above the top of the socket). The optical sections were taken from directly above the socket, along the long axis of the cut-off hair. Images on the left were obtained with Koehler illumination, and the images on the right were obtained in fluorescence confocal mode. Each horizontal pair was obtained at the same image plane, with the deepest section nearest the base of the hair in the bottom pair of panels (Figure 2A4 and 2B4). The filiform hair is shown most clearly in the confocal series on the right: the hair, which is strongly autofluorescent, appears in cross-section as the bright thick ellipsoid in the center of the socket septum. The filiform hairs are hollow, and the lumen in this hair appears as the dark compressed-D-shaped spot in the center of the hair. The rim of the socket septum appears as another thinner bright ellipsoidal ring around the hair. That ellipsoidal rim is also clearly visible in the DIC images in the left panels. For this hair/socket structure, the major axis of the ellipsoidal socket rim and hair base is approximately 30 degrees counterclockwise from the vertical with respect to the image frame, so that the direction of the hair movement would be along a line rotated approximately 60 degrees clockwise from the vertical.

#### Asymmetric socket septum

Both sets of panels in Figure 2 also show asymmetries in the appearance of the socket base. In panels A (the DIC images), there is a bright “tick” feature extending from the right side of the short axis of the ellipsoidal septum cavity. In panels B (the auto-fluorescent confocal image), there is a corresponding feature: a dark notch extending from the ellipsoidal cavity into the rim of the socket. We interpret the tick seen in the DIC images as arising from the contrasting DIC images of the opposing edges of the tissue on either side of the notch. This notch feature was seen in every filiform socket we imaged, and was clearly visible using microscope objectives with magnification as low as 20×. This is the critical, essential morphological feature upon which our subsequent analysis is based: as we will show below, this feature identifies the hair's excitatory deflection direction.

These image stacks also show an additional and important asymmetric feature in the base of the hair itself: the *ecdysial canal*. Note the additional smaller dark spot that bulges out from the lumen in the bottom right panel (2B4), and then diverges away from the lumen toward the notch feature in the higher sections. This small dark spot corresponds to the ecdysial canal, which extends from the base of the hair lumen to terminate as a hole in the surface of the hair several sections above the one shown in panels 2A1 and 2B1. The terminal dendritic segment of the mechanosensory receptor cell is anchored to the hair shaft within the ecdysial canal. The most important point from the perspective of our study is that previous studies using electron-microscopic and neurophysiological studies have demonstrated that the ecdysial canal opening is in the excitatory direction of the hair's movement [22], [27], [28].

**Figure 3:**
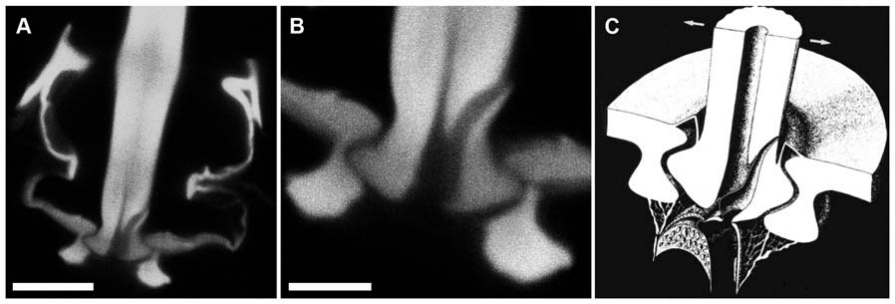
Anatomy of the base of a typical filiform mechanosensory hair in its socket. A. Confocal image of a segment of a filiform hair within its socket, which was removed intact from a fixed cercus. B. A higher-magnification image of the base region, showing the ecdysial canal extending from the base of the hair up to its exit point on the right side of the hair above the socket base. C. Schematic diagram of the base of a filiform hair and socket derived from detailed electron microscopic analysis, illustrating the configuration of the ecdysial canal, reproduced from [31]. Images in panels A and B were obtained with a Zeiss Neofluar 100× oil immersion objective with NA of 1.0. Scale bars: A: 5 microns, B: 2 microns.

Figure 3 illustrates the anatomy of the base of a typical filiform mechanosensory hair in its normal configuration in a socket, showing a different view of the ecdysial canal. Panel 3A is a confocal image of a segment of a filiform hair within its socket, which was removed intact from a fixed cercus. This image is taken from the side of the hair and socket, rather than from the top as in Figure 2. Panel B of Figure 3 is a higher-magnification image of the base region, and clearly shows the ecdysial canal extending from the base of the hair up to its exit point on the right side of the hair above the socket base.

Panel C is a schematic diagram of the base of a filiform hair and socket derived from detailed electron microscopic analysis, illustrating the configuration of the ecdysial canal, reproduced from [31]. There is a direct correspondence between the ecdysial canal identified in earlier EM studies diagrammed in Figure 3C, and the dark channel seen from the side in panels 3A and 3B, and in cross section in the confocal panels of Figure 2.

We examined twenty-three hairs in this manner, and in every case the ecdysial canal exited the hair in the direction of the notch feature. Thus, the position of this notch feature, which is clearly visible with conventional light microscopic observation, is a definitive indication of the excitatory movement direction of the associated filiform hair.

### The notch feature is visible in sockets from which the filiform hairs have been removed

**Figure 4:**
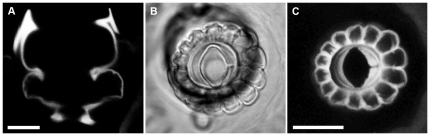
Light microscopic images of two typical filiform hair sockets after the hairs had been removed. A. Side view of a socket that had been removed from a cercus. B,C. Top views of a second socket, imaged with DIC and confocal illumination modes, respectively. Note the “notch feature” on the right side of each image, corresponding to the direction of movement that elicits excitation of the mechanoreceptor. Images were obtained with a 40×1.25 NA oil immersion objective. Scale bars: A: 5 microns, B,C: 20 microns.

Because of the precise correspondence between the cavity in the socket septum and the hair base, a hair's excitatory movement direction can also be determined by direct observation of the socket septum cavity after the hair has been removed. Light microscopic images of the sockets of two typical filiform hairs are shown in Figure 4. The hair has been plucked from these sockets, as described in the Methods section. Figure 4A shows a side view of a socket that was removed intact from a cercus after it's hair had been plucked out. This image demonstrates that the structure of the socket septum and hair-base cavity remains intact (compare to Figure 3A). Figures 4B and 4C show top views of a second socket, imaged with DIC and confocal illumination modes, respectively. The intrinsic autofluorescence of the cuticle enables a clear visualization of the socket structure in the confocal image. The notch feature is clearly visible in both images. For this second socket shown in panels B and C, the major axis of the septum cavity is approximately vertical with respect to the image frame, so that the direction of the hair movement would be along the horizontal direction of the frame. Furthermore, the excitatory direction is to the right, toward the notch feature.

### The lengths of filiform hairs can be estimated from light microscopic images of the hair sockets

**Figure 5:**
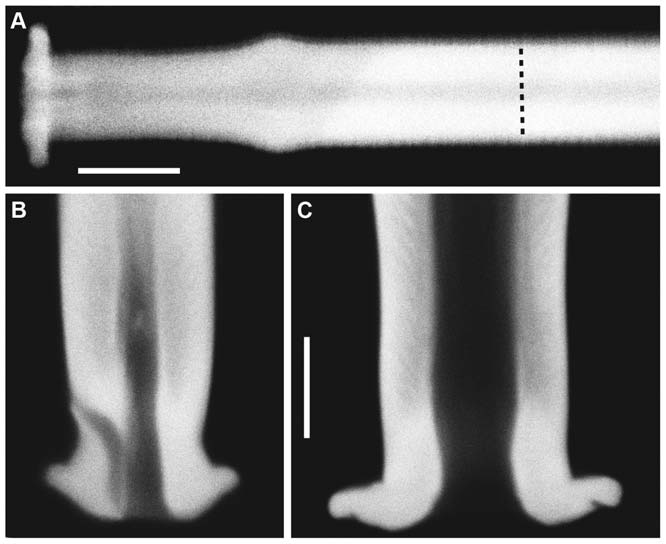
Optical sections through the long axes of isolated filiform hairs near their basal ends. A. Segment including the base of the hair (on the left of the image). The hair is oriented with the exit of the ecdysial canal oriented directly out of the image plane. The ecdysial exit canal is difficult to distinguish, and is a dark patch near the base of the hair. The bulge in the hair corresponds to the position at which the hair passes through the top aperture of the cuticular socket (as shown in Figure 3A). The location at which the diameter measurement was taken from of this hair (and other hairs) is indicated with a dashed line. B,C. higher-magnification images of the base of two filiform hairs of nearly identical length. B. An optical section directly through the central lumen of the hair at the point corresponding to the minor axis of the ellipsoidal base flange. The hair cross section is also ellipsoidal at this point below the bulge, and this image orientation presents the narrowest projection of the hair. The ecdysial canal can be seen as a small dark band rising up from the left side of the base. C. Section through the center of a different hair of nearly equal length to the one shown in panel B, but from a viewing angle rotated 90 degrees from the image in panel B. This section shows the full length of the major axis of the base flange, through the articular pegs. This image orientation also presents the widest projection of the ellipsoidal hair shaft itself. Our measurements were of the major axes of the hair base flanges, corresponding to panels A and C. Scale bars: A: 10 microns, B,C: 5 microns.

**Figure 6:**
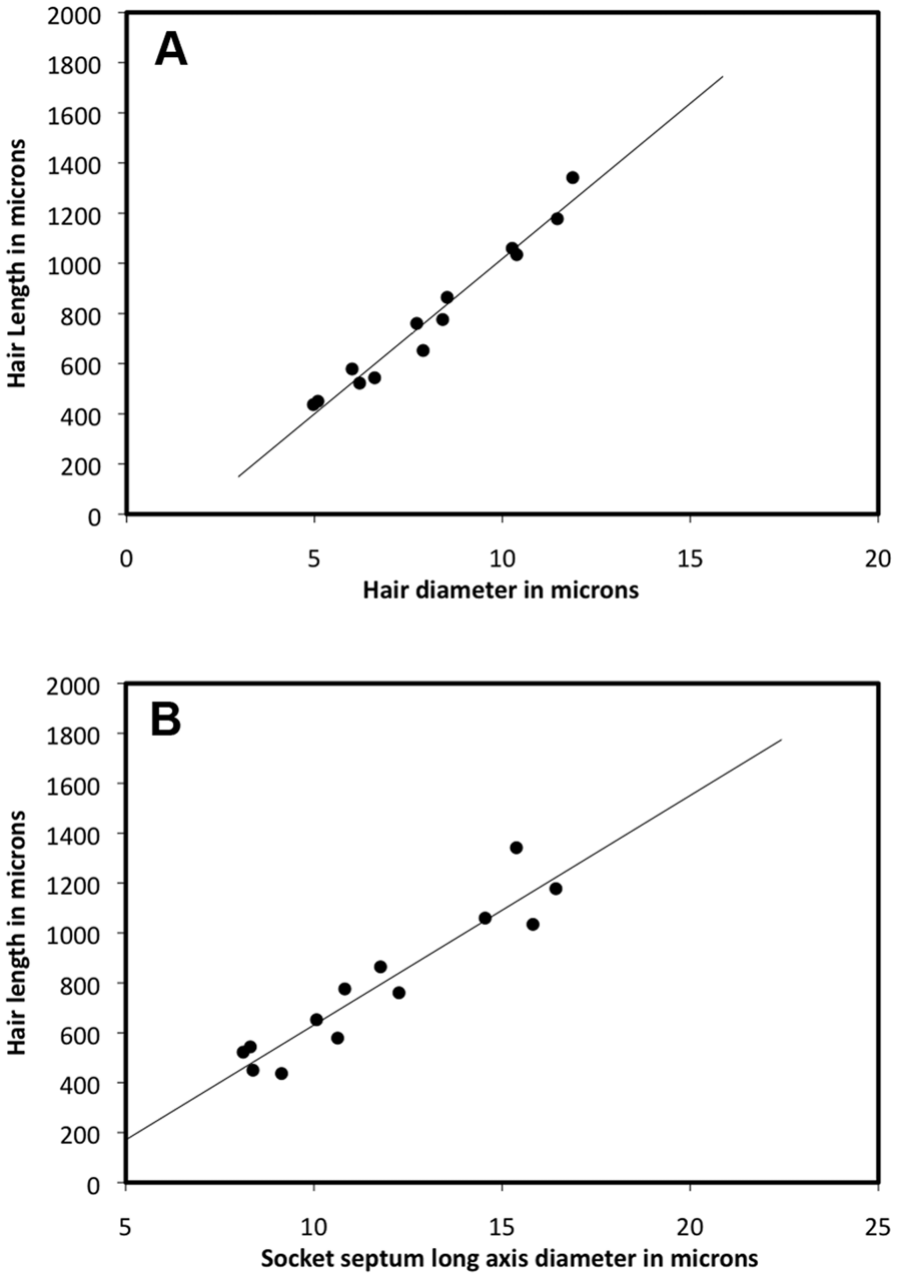
Relationships between hair length, hair diameter and hair base diameter. A. Plot of the hair length vs. hair diameter at the location indicated with **d** in Figure 5A for a sample of 13 hairs. The data lie along a line defined by **l** = 124**d**–219, where **l** and **d** signify hair length and diameter in microns (R^2^ = 0.97). B. Plot of the lengths of the thirteen filiform hairs vs. the dimension of the long axis of the ellipsoidal base of the socket for each of these hairs. The data lie along a line defined by **l** = 92**m**–289, where **l** and **m** signify hair length and major axis of the base in microns (R^2^ = 0.89).

Previous studies in other species have demonstrated a systematic relationship between the length of a cercal filiform hair and its diameter near the base [5], [18], [32]. We determined the relationship in *Acheta domesticus* by direct microscopic measurement of the lengths and diameters of a sample of thirteen filiform hairs that had been plucked from the cerci of three different animals. Figure 5A shows a segment of a filiform hair including its base. The hair had been plucked from a cercus as described in the Methods section. The point at which the diameter was measured is indicated with a dashed line. This point was chosen for consistency across all hairs in the sample: it is at a distance beyond the bulge equal to the distance between the base and the bulge. The bulge in the hair corresponds to the position at which the hair passes through the top aperture of the cuticular socket (as shown in figure 3A). The hair has a circular cross section and a uniform diameter from this bulge outward, whereas the hair cross section is ellipsoidal and its diameter is non-uniform between the bulge and the base (see Figure 2). Figure 6A shows a plot of the length vs. diameter for these hairs. The data lie along a line defined by **l** = 124**d**–219, where **l** and **d** signify hair length and diameter in microns (R^2^ = 0.97). Note that this function differs from the functions determined for other species of crickets in two respects: it is a linear function, and the coefficients are different.

We also determined the relationship between the length of a filiform hair and the dimensions of the major axis of the ellipsoidal socket base. Figures 5B and C show higher-magnification images of the base of two filiform hairs of nearly identical length. Panel B shows an optical section directly through the central lumen of the hair at the point corresponding to the minor axis of the ellipsoidal base flange. The hair cross section is also ellipsoidal at this point, and this image orientation presents the narrowest projection of the hair (see Figures 2B1–4). The ecdysial canal can be seen as a small dark band rising up from the left side of the base. Figure 5C shows a section through the center of a different hair of nearly equal length to that shown in panel B, but from a viewing angle rotated 90 degrees from the image in panel B. This section shows the full length of the major axis of the base flange, through the articular pegs. This image orientation also presents the widest projection of the ellipsoidal hair shaft itself. The ecdysial canal cannot be seen, because it is oriented straight out of the image plane well out of the depth of field of this optical section. Our measurements were of the major axes of the hair base flanges, corresponding to panels B and C.

Figure 6B shows a plot of the lengths of the thirteen filiform hairs vs. the dimension of the long axis of the ellipsoidal base of the socket for each of these hairs. The data lie along a line defined by **l** = 92**m**–289, where **l** and **m** signify hair length and major axis of the base in micrometers (R^2^ = 0.89).

The linearity with high R^2^ value for this relation is the basis for a reasonably reliable means for estimating the length of the filiform hair associated with a socket from which that hair had been plucked, based on observation of the dimensions of the empty socket septum. Specifically, since the cavity in a socket septum corresponds precisely to the shape of the flange at the base of the hair that had been plucked from that socket, the length of the filiform hair can be estimated from the length of the major axis of the empty socket septum cavity.

### Large-scale pattern of the lengths and directions of filiform hairs on the cerci

**Figure 7:**
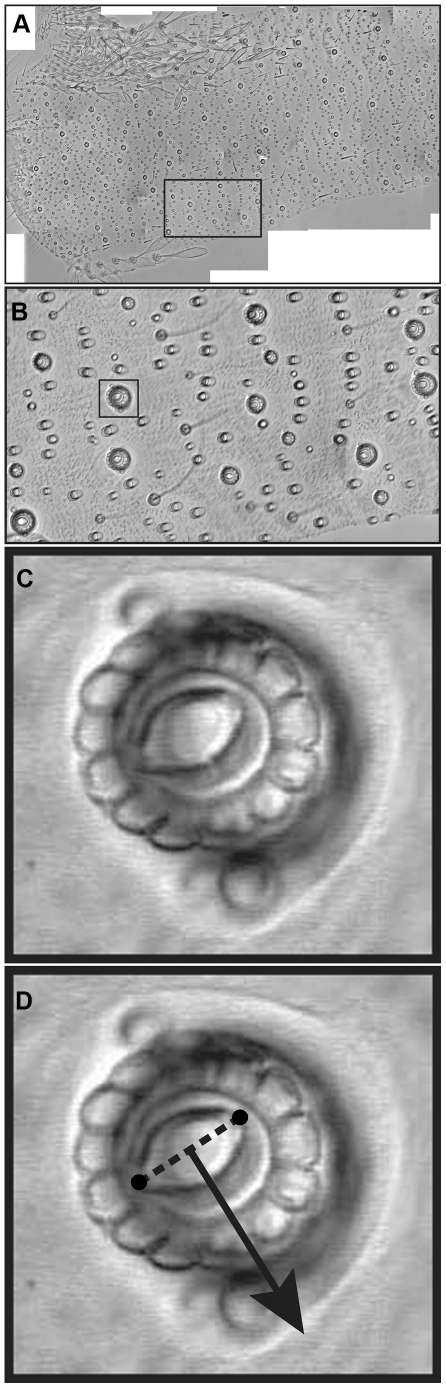
Successively higher magnification views of a collage of overlapping images captured from one cercus fillet. A. Portion of the collage that stretched from the attachment point at the base of the cercus (on the left of this image) to approximately 25% of the total length of the cercus. B. Higher-magnification view of the area in A indicated by the rectangular box. C. An even higher magnification view of the single socket indicated with the square box in B. The socket septum cavity and notch feature are clearly visible at this resolution. D. Illustration of the standard data collection points for this socket. The two small circles joined with a dashed line indicate our measurement of the long axis diameter of the socket septum cavity (and hence, the pivot axis and an indication of hair length), and the arrow represents the direction of filiform hair movement that would cause excitation. Images were obtained with a 20×0.5NA dry objective in DIC mode. Scales: width of bounding panel boxes in A: 33 mm, B: 740 microns, C,D: 66 microns.

In order to record the positions, orientations and major axis dimensions of filiform hair sockets in their natural configuration, we observed flattened “fillets” of cerci from which all filiform hairs had been removed. As described in the Methods section, the cerci were cut along their long axes, flattened onto microscope slides, and imaged with DIC optics at adequate magnification to resolve the notch feature and measure the major axis of the sockets. Figure 7 shows successively higher magnification views of a collage of the 43 individual overlapping images captured from one cercus fillet. Panel A of Figure 7 shows a portion of the collage that stretched from the abdominal attachment point at the base of the cercus (on the left of this image) to approximately 25% of the total length of the cercus. Panel B is a higher-magnification view of the area in panel A indicated by the rectangular box. Panel C shows an even higher magnification view of the single socket indicated with the square box in panel B. The socket septum cavity and notch feature are clearly visible at this resolution. For three different cercal fillet collages, the length and orientation of the long axis of every socket septum was recorded, along with an indicator of the side of the socket that showed the notch feature. Panel D illustrates how anatomical data was recorded from this socket: the two small circles joined with a dashed line indicate our measurement of the long axis diameter of the socket septum cavity (and hence, the pivot axis and an indication of hair length), and the arrow represents the direction of filiform hair movement that would cause excitation.

**Figure 8:**
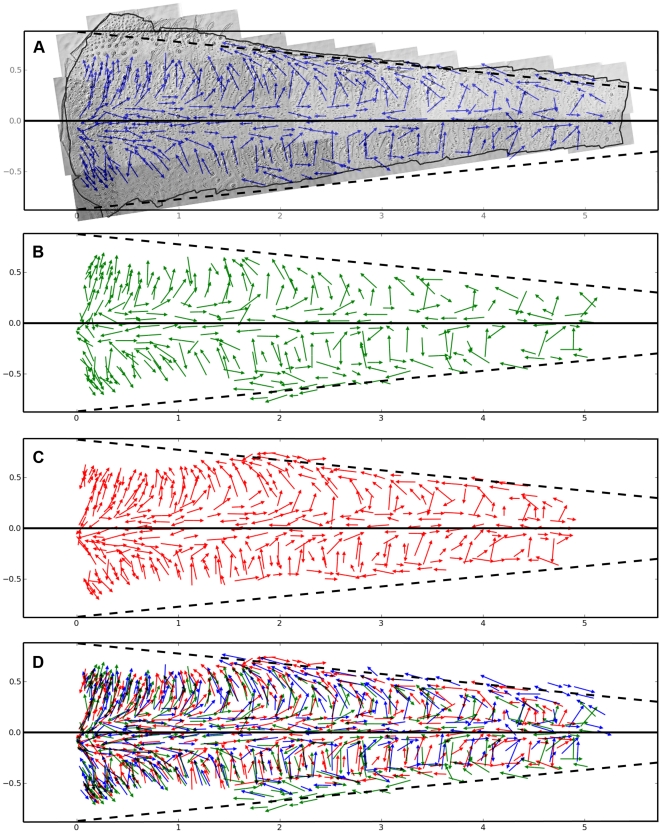
Filiform hair parameters for three cercal filet preparations, color coded by preparation. A. Complete collage of all 43 micrographic gray-scale images for an entire cercal filet preparation. The collage spanned from the base of the cercus to 50% of the total cercus length. The outline of the tissue has been drawn over the collage for clarity. The X and Y axis labels of the bounding box in this and all other panels in this figure are in millimeters. Note that the collage has been oriented within the box so that the lateral longitudinal axis of the cercus is defined as the X axis (i.e., Y = 0), indicated with a solid line. The medial longitudinal axis is indicated with the dashed lines near the top and bottom edges of the filet preparation: these lines both represent the same axis, and would wrap around to superimpose on one another forming the conical geometric form of the cercus. Each blue arrow corresponds to anatomical measurements from a single hair socket. The center point of each vector corresponds to the location of the corresponding filiform hair socket, and the length of the vector is proportional to the length of the major axis of the socket septum (and, thus, the length of the hair). The excitatory direction of hair movement is represented by the direction of each arrow. B,C. Vectors representing the anatomical data for sockets in two more filet preparations, covering exactly equivalent segments of the cerci, without the gray-scale collage images. D. Composite of the data from all three preparations in panels A–C. The vectors for each of the top three panels are plotted in a different color; that color coding is maintained in the composite. Note that some of the vectors appearing near the top and bottom boundaries of the three different preparations do not overlap. This arises from an experimental artifact: the cuts along the medial longitudinal axes of the three different cerci were not along exactly the same lines.

The complete collage of all 43 micrographic gray-scale images for an entire cercal filet preparation is shown in panel A of Figure 8. The entire collage spanned from the base of the cercus to 50% of the total cercus length. The outline of the tissue has been drawn over the collage for clarity. The X and Y axis labels of the bounding box are in millimeters. Note that the collage has been oriented within the box so that the lateral longitudinal axis of the cercus is defined as the X axis (i.e., Y = 0), indicated with a solid line. The medial longitudinal axis is indicated with the dashed lines near the top and bottom edges of the filet preparation: these lines both represent the same axis, and would wrap around to superimpose on one another forming the conical geometric form of the cercus. Superimposed on the collage are 300 blue arrows. Each arrow corresponds to anatomical measurements from a single hair socket, recorded as illustrated in Figure 7D. The center point of each vector corresponds to the location of the corresponding filiform hair socket, and the length of the vector is proportional to the length of the major axis of the socket septum (and, thus, the length of the hair). The excitatory direction of hair movement is represented by the direction of each arrow.

Panels B and C of Figure 8 plot the vectors representing the anatomical data for sockets in two more filet preparations from two additional crickets, covering equivalent segments of the cerci, without the gray-scale collage images. The bottom panel D is a composite of the data from all three preparations in panels A–C. The vectors for each of the top three panels are plotted in a different color; that color coding is maintained in the composite shown in panel D. Visual inspection of panel D shows a high degree of similarity of all three preparations with respect to the local density, length distributions, and “banding” of the directional selectivities of the hairs. The banding has been noted in earlier reports [1]–[4], [23]–[25]. More quantitative analyses of these organizational aspects of the filiform array are presented below. Note in panel D that some of the vectors appearing near the top and bottom boundaries of the three different preparations in panels A–C do not overlap. This arises from an experimental artifact: the cuts along the medial longitudinal axes of the three different cerci were not along exactly the same lines. This non-uniformity is accounted for in subsequent figures and analyses.

Table 1 summarizes data related to the number of sockets we recorded in our three samples. All data used for this table and for all subsequent figures are provided as supporting information (see spreadsheet S1 online). In Table 1, specimen numbers 1, 2 and 3 correspond to the vector data sets shown in panels A, B and C of Figure 8, respectively. The total number of sockets is shown in the first column. The number (and fraction) of hairs in each of three length categories are shown in the right three columns. The ranges for these length categories were chosen to subdivide the grand average (335 sockets/specimen) into three approximately equal fractions. These data indicate that there is considerable inter-animal variation in the number and length distributions of the hairs.

**Table 1:** Number of filiform hair sockets in basal 50% of three cerci.

Specimen	Total sockets	Sockets for short hairs (<775 µ) Number (fraction)	Sockets for medium hairs (775–1200 µ) Number (fraction)	Sockets for long hairs (>1200 µ) Number (fraction)
1	300	54 (0.18)	122 (0.41)	124 (0.41)
2	293	72 (0.25)	96 (0.33)	125 (0.43)
3	412	216 (0.52)	115 (0.28)	81 (0.20)
Average	335	114 (0.34)	111 (0.33)	110 (0.33)

Specimen numbers 1, 2 and 3 correspond to the vector data sets shown in panels A–C of Figure 8, respectively.

**Figure 9:**
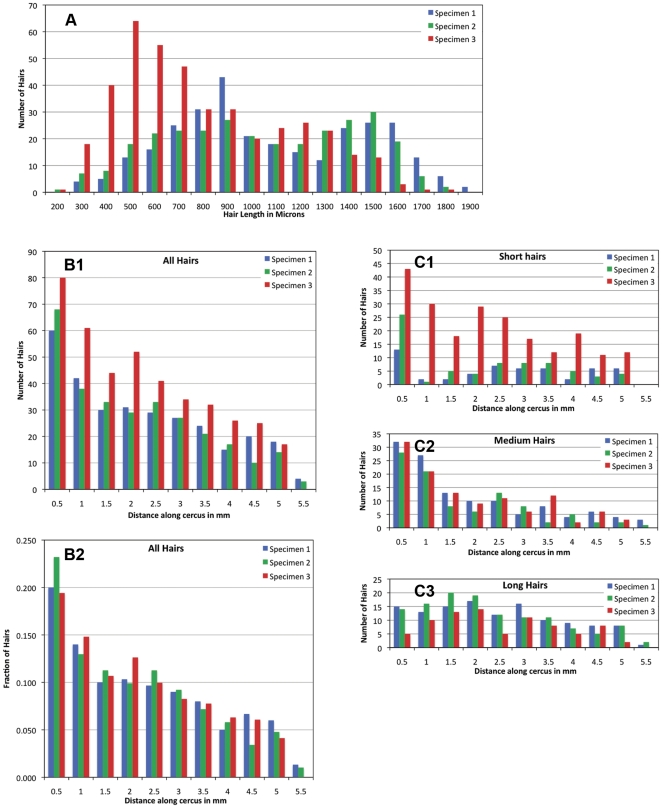
Distribution of filiform mechanoreceptors as functions of distance and length. A. Histograms of the number of hairs as a function of hair length. The data from each specimen is shown in a different color, maintaining the same color scheme used in figure 8. B1,B2: Histograms of the density of hairs as a function of position out along the cercus. All panels maintain the same color coding scheme used in panel A. B1. Raw data for the counts of all hairs in bins at successive distances away from the base of the cerci, for all three preparations. B2. Same data as in B1, but with the amplitudes of the bin counts for each specimen scaled by the total number of hairs in that preparation. C1–C3: Histograms of the density of hairs as a function of position out along the cercus, subdivided by hair length ranges. C1. Histogram of the raw (*i.e.*, un-scaled) data for the sockets corresponding to the short hairs (shorter than 775 microns.) C2. Histogram of the raw data for the sockets corresponding to the medium hairs (775–1200 microns.) C3. Histogram of the raw data for the sockets corresponding to the long hairs (longer than 1200 microns.)

This data is shown more graphically in Figure 9A, which presents histograms of the number of hairs as a function of hair length. The data from each specimen is shown in a different color, maintaining the same color scheme used in Figure 8. Note that the distributions for specimens 1 and 2 (blue and green) are very similar in general form, but the distribution for specimen 3 is very different from the other two: it has a smaller proportion of long hairs, and a much greater proportion of shorter hairs. We note that this difference is not likely to be due to any differences in experimental or data collection procedures: all procedures were identical for the three specimens, within a very short time window. However, we note that the three specimens came from two different (sequential) shipments of crickets: specimens 1 and 2 were from the same shipment, and specimen 3 was from the preceding shipment.

Panels B1 and B2 of Figure 9 are histograms of the density of hairs as a function of position out along the cercus. All panels maintain the same color scheme used in Figures 8 and 9A. Panel 9B1 shows data for all hairs from all three preparations. The general trends for the three different specimens are similar, with the data for specimen 3 (in red) reflecting the greater number of hair sockets. Panel 9B2 plots this same data, but with the amplitudes of the bins for each specimen scaled by the total number of hairs in that preparation. Here, the similarity in the density distributions across preparations is very clear.

Although the general density distribution trends are very similar, panels 9C1 through 9C3 demonstrate that the relative distributions of hairs as a function of their length ranges differ significantly between the three specimens, with the biggest disparity again being between specimen 3 (in red) and the other two specimens. Panel 9C1 is the histogram of the raw (*i.e.*, un-scaled) data for the sockets corresponding to the short hairs (shorter than 775 microns, as defined in Table 1). Histograms for the medium (775–1200 microns) and long (longer than 1200 microns) hairs are shown in panels 9C2 and 9C3, respectively. Although the histograms for the medium length hairs are very similar across all three specimens, the data for the short and long hairs is substantially different for specimen 3 versus the other two specimens. Implications of the inter-animal variability are considered in more detail in the Discussion section.

**Figure 10:**
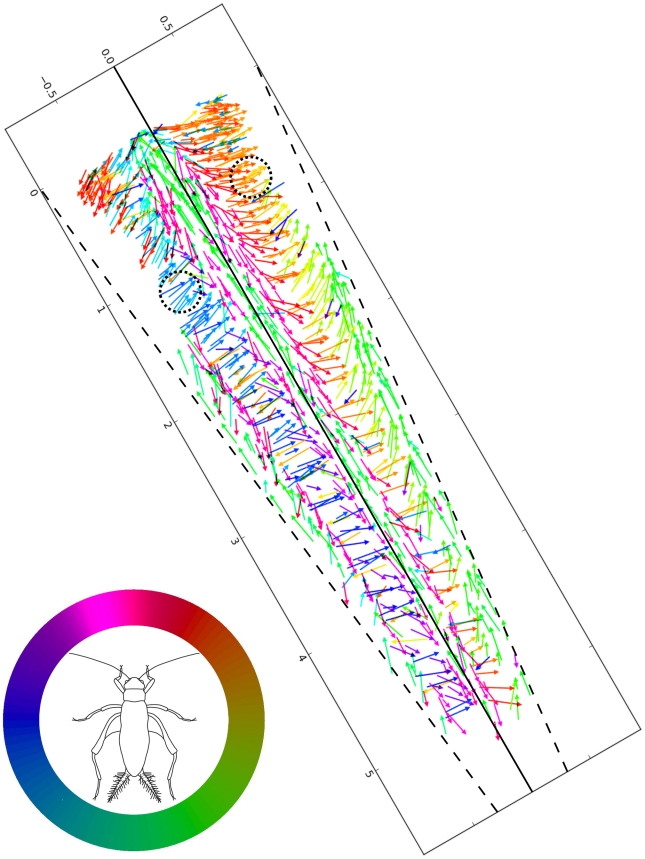
Filiform hair parameters for three cercal filet preparations, color coded by hair movement direction. This combined data set uses all vectors as presented in Figure 8D, but with the vectors color coded by the direction of the hair movement rather than by preparation. The X and Y axis labels of the bounding box are in millimeters. For clarity, the panel is rotated into the natural orientation of the right cercus with respect to the cricket's body, if the cricket were facing upward (as indicated by the line drawing of a cricket, inset within the color legend). Note that the direction colors are referenced to the body axis of the cricket, rather than to the long axis of the cercus, as indicated by the circular reference legend. Note that the color of each vector is coded according to the direction of the corresponding air current stimulus that would push the hair in it's optimal direction, rather than to the hair's optimal movement direction: the indicated vector color is actually the compass direction opposite to the hair's excitatory movement direction. For example, a green vector indicates an excitatory movement direction directly along the long axis of the right cercus toward the animal's body, stimulated by air currents directed at the cricket from the right rear. The two dashed circles near the top of the vector panel indicate vectors that point in approximately the same direction, but are coded with colors that are diametrically opposed. This is due to the fact that the arrows in the two different regions are on different surfaces of the cercus.

Figure 10 shows the same (combined) data set as presented in Figure 8D, but with the vectors color coded by the direction of hair movement rather than by preparation. For clarity, the panel is rotated into the natural orientation of the right cercus with respect to the cricket's body, if the cricket were facing upward (as indicated by the line drawing of a cricket, inset within the color legend). Note that the direction colors are referenced to the body axis of the cricket, rather than to the long axis of the cercus, as indicated by the circular reference legend at the bottom left. It is important to note that the color of each vector is coded according to the direction of the corresponding air current stimulus that would push the hair along it's optimal excitatory movement direction, rather than to the hair's optimal direction itself. I.e., the indicated vector color is actually the compass direction opposite to the hair's excitatory movement direction. 0 degrees is defined as a stimulus directed toward the animal from the front, with a positive-going increase in angle representing a clockwise rotation around the animal's body as viewed from above (e.g., 90 degrees corresponds to a stimulus from the right). For example, a green vector indicates an excitatory movement direction directly along the long axis of the right cercus toward the animal's body, stimulated by air currents directed at the cricket from 150 degrees. This stimulus-referenced color coding convention was chosen for consistency with previous publications by us and other authors over the last two decades.

Careful inspection of the vector colors in this figure will reveal an aspect that might, at first, seem to be an error in color assignment. Consider the arrows indicated to lie within the two regions within the dashed circles near the top of the panel. The vectors point in approximately the same direction, but are coded with colors that are diametrically opposed. This is due to the fact that the arrows in the left circle are actually on the dorsal surface of the cercus, and those in the right circle are on the ventral surface. If this flattened filet were wrapped around to re-construct the true conical geometry by joining the two dashed lines corresponding to the medial longitudinal axis, then the red-orange vectors in the upper-right dashed circle would be on the lower surface of the cercus, and would actually orient in the opposite direction from that shown here on the flattened filet. The blue vectors in the left dashed circle are on the dorsal surface, and the color coding and vector directions therefore appear to agree with one another. All color coding, and all subsequent calculations related to the distribution of hair directions, take this conical geometry into account.

**Figure 11:**
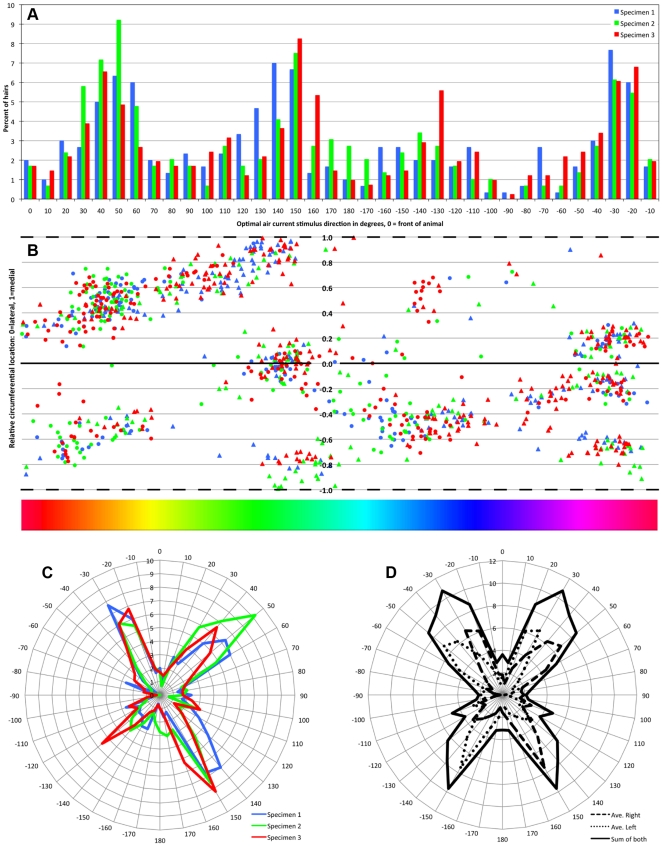
Representations of the number of hairs as a function of excitatory stimulus direction. A. Histogram of the number of hairs as a function of excitatory stimulus direction from all three preparations, with data from each preparation shown in a different color (maintaining the same color scheme used in Figures 8 and 9), and with the amplitudes of the bins for each specimen scaled by the total number of hairs in that preparation (as in panel 9B2). The direction labeled on the X axis is the direction of the optimal stimulus to move the hairs, referenced to the body axis of the animal, with 0 degrees defined as a stimulus directed toward the animal from the front. B. Different representation of the same data shown in A, related to the patterns of banding of directional selectivities around the circumference of the cerci. The X axis in panel B is the same as for panel A: it is the direction of the optimal stimulus to move the hairs, referenced to the body axis of the animal. The color bar at the base of panel B provides a visual cue to these angle values, and is referenced to the color wheel in figures 10 and 13. The Y axis is the radial location around the circumference of the conical cercal surface, with zero (solid line) corresponding to the lateral face and 1 (top and bottom dashed lines) corresponding to the medial “cut lines” as shown in Figures 8 and 10. The ventral hemi-cone is on the top half of this scatter plot, and the dorsal hemi-cone is on the bottom half. Each point in this scatter plot corresponds to a single socket from one of the three specimens, maintaining the specimen-based color coding used in Figures 8, 9 and 11A. C. Same data used for panel A, but represented as a polar plot. D. Calculations based on the data presented in Figure 11C, representing an estimate of the entire distribution of hairs on the basal 50% of *both* cerci. The plot with a dashed line is the combined data from panel 11C, and the plot with the dotted line is the reflection of the dashed curve around the body axis. This dotted curve is an estimate of the mean distribution of hairs on left cerci. The dark solid curve is the sum of both curves, and estimates the total sensory input to which the cricket would have access, from the basal halves of both cerci.

Representations of the number of hairs as a function of excitatory stimulus direction are shown in Figure 11. Panel 11A shows data from all three preparations as a histogram, with data from each preparation shown in a different color (maintaining the same color scheme used in Figures 8 and 9), and with the amplitudes of the bins for each specimen scaled by the total number of hairs in that preparation (as in panel 9B2). As in Figure 10, the direction labeled on the X axis is the direction of the optimal stimulus to move the hairs, referenced to the body axis of the animal, with 0 degrees defined as a stimulus directed toward the animal from the front. The distributions of directional selectivities for the three specimens are similar to one another: all are extremely non-uniform, and all have four major peaks centered on approximately the same directions. This basic four-peaked distribution was reported earlier [4], though the details of the distributions reported here differ in the relative amplitudes of the peaks.

Figure 11B shows a different representation of this same data, related to the patterns of banding of directional selectivities around the circumference of the cerci. The X axis in panel 11B is the same as for panel 11A: it is the direction of the optimal stimulus to move the hairs, referenced to the body axis of the animal. The Y axis is the radial location around the circumference of the conical cercal surface, with zero corresponding to the lateral face and 1 corresponding to the medial “cut line” in Figures 8 and 10. The ventral hemi-cone is on the top half of this scatter plot, and the dorsal hemi-cone is on the bottom half. Each point in this scatter plot corresponds to a single socket from one of the three specimens, maintaining the specimen-based color coding used in Figures 8, 9 and 11A. The fact that the data points are clustered with respect to both axes corresponds to the non-uniformity in two aspects of their functional distribution. The clustering along the X axis corresponds to the non-uniformity in directional selectivities, as discussed in the preceding paragraph, and as is shown in Figure 11A. Specifically, note that the data point clusters in panel 11B line up directly under the peaks in the histogram of panel 11A: these aspects of the two plots arise from this same organizational feature of the receptor array. The clustering of the data points along the Y axis in panel 11B, however, are not reflected in the histogram of panel 11A, and arise from a different aspect of the organization of the array: the clustering of mechanosensors having similar directions into longitudinal bands along the long axis of the cerci. Specifically, the shape of the histogram in 11A could have been obtained if there were no clustering in the Y dimension (i.e., around the cercal circumference). The possible functional significance of this banding is considered in the Discussion.

Panel 11C is the same data used for panel 11A, but represented as a polar plot, to show the distributions more intuitively. It is clear from this polar plot that the peaks in the directional selectivity distributions correspond to the longitudinal and transverse axes of the cerci, confirming the qualitative impression from a visual inspection of the vectors in Figures 8 and 10. Panel 11D presents the results of calculations based on the data presented in Figure 11C, representing an estimate of the entire distribution of hairs on the basal 50% of *both* cerci. The plot with a dashed line is the combined data from panel 11C, and the plot with the dotted line is the reflection of the dashed curve around the body axis. This dotted curve is an estimate of the mean distribution of hairs on these left cerci. The dark solid curve is the sum of both curves, and estimates the total sensory input to which the cricket would have access, from the basal halves of both cerci.

### Characteristics of the “cercal fovea”

**Figure 12:**
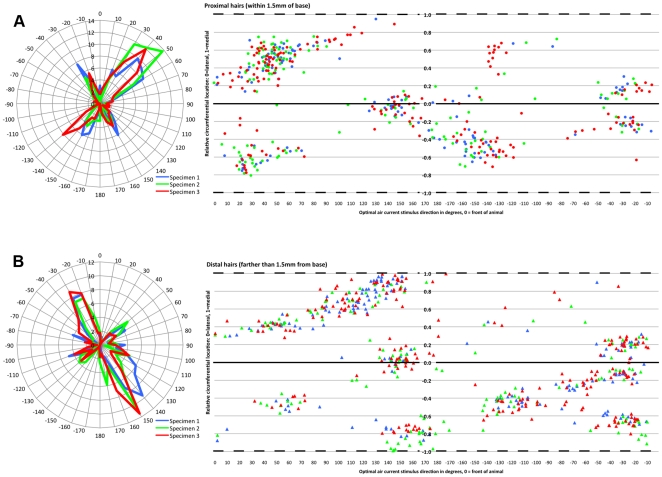
Differences in ratios of transverse to longitudinal filiform hairs in two regions of the cerci. A. Polar plot of the number of receptors within the basal 1.5 mm “cercal fovea”, as a function of their directional selectivity. Data for each of the three specimens is plotted in a different color, maintaining the specimen-based color coding used in earlier figures. To the right of the of the polar plot is a scatter plot for the corresponding data, presented as in panel 11B: the X axis is the direction of the optimal air current stimulus, and the Y axis is the radial location around the circumference of the conical cercal surface, with zero (solid line) corresponding to the lateral face and 1 (dashed lines) corresponding to the medial “cut lines” shown in Figures 8 and 10. B. Similar plots for the data corresponding to all receptors beyond the basal 1.5 mm region.

As shown in Figure 9, the density of receptors is much greater within the 1.5 mm region near the base of each cercus than it is farther out along the cercus. Visual inspection of Figures 8 and 10 indicate that at least one more aspect of the functional organization of the filiform receptor array also differs significantly between the basal 1.5 mm region of the cercus and the more distal region: the ratios of longitudinal to transverse hairs. The difference in ratios of transverse to longitudinal filiform mechanosensors in these two regions are illustrated in Figure 12. Figure 12A uses a subset of the data plotted in panel 11C: it is a polar plot of the number of receptors as a function of their directional selectivity within the basal 1.5 mm of the cerci. Figure 12B plots the hair numbers vs. directional selectivity for receptors in the portions of the cerci beyond 1.5 mm. To the right of each of these polar plots is a scatter plot for the corresponding data, presented as in panel 11B. It is clear from these plots that there are significantly more transverse hairs in the basal 1.5 mm of the cerci specimens. This, in turn, yields different overall directional selectivity curves for each region.

**Figure 13:**
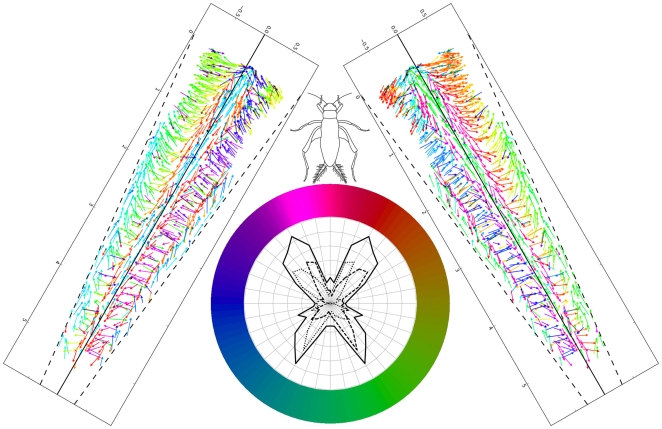
Graphical summary of the major results of this study. The panel of vectors on the right shows the combined data sets for hairs on the three specimens used for our study, with all vectors color coded by the optimal stimulus direction for hair movement. The panel is rotated into the natural orientation of the right cercus with respect to the cricket's body, if the cricket were facing upward (as indicated by the line drawing of a cricket at top center). The panel of vectors on the left is an estimate of the combined distribution of sockets from three left cerci, assuming approximate mirror symmetry. The vector colors represent the optimal direction of air currents that would move the corresponding hair, referenced to the body axis of the cricket as defined by the circular reference legend at bottom center. The circular reference legend for vector color assignment contains an inset that presents the estimate of the entire distribution of hairs on the basal 50% of *both* cerci, as shown in Figure 11D.

Figure 13 is a graphical summary of the major results of this study. The panel of vectors on the right are identical to the group on the right of Figure 10, and shows the combined data sets for hairs on the three specimens used for our study, with all vectors color coded by the optimal stimulus direction for hair movement. As in Figure 10, the panel is rotated into the natural orientation of the right cercus with respect to the cricket's body, if the cricket were facing upward (as indicated by the line drawing of a cricket at top center). Also as in Figure 10, the vector colors represent the optimal direction of air currents that would move the corresponding hair, referenced to the body axis of the cricket as defined by the circular reference legend at bottom center. The panel of vectors on the left is an estimate of the combined distribution of sockets from three *left* cerci, assuming approximate mirror symmetry. This image was obtained by mirror-reflecting the dataset for he right cercus vectors, and re-assigning the vector color codes appropriately. The circular reference legend for vector color assignment contains an inset that presents the estimate of the entire distribution of hairs on the basal 50% of *both* cerci, as shown in Figure 11D.

## Discussion

The global patterns of the filiform hairs' directional selectivities and packing densities on the cerci of *Acheta domesticus* display remarkable structure, and were first described qualitatively by Palka and his colleagues [1]. It was noted in that early study that a) the directionality of hairs is organized in bands along the long axis of the cerci, b) there is a systematic rotation in the movement directions of the hairs around the circumference of each cercus, and c) there is a non-uniform distribution of hair densities along the length of the cercus, with greater density nearer the base than near the tip. These organization features of the filiform hair array in this and other closely related species have been verified in several subsequent studies [3], [4], [18], [23]–[25], and one of these studies also documented how the distributions of preferred hair directions from the two cerci result in a non-uniform distribution containing distinct peaks [4].

All of those observations are confirmed and characterized quantitatively in the studies presented here. Our graphs in Figure 11A and 11B for the non-uniform distribution of hair directionality and circumferential distributions are generally similar to those published earlier [4]. The differences between our data and measurements published in that report are most likely due to our larger sample size (1005 *vs.* 246 hairs in the earlier study), our more accurate means for determination of excitatory directionality, and our inclusion of hairs of all length ranges. The earlier report was restricted to a randomly selected set of long and intermediate length hairs (most over 1 mm, none shorter than 400 microns), whereas our study attempted to capture all hairs on the basal half of three cerci specimens.

Beyond contributing a more quantitative characterization of the filiform afferent array, the results presented here extend and challenge the current understanding of the system in several fundamental respects. Specifically, we present new results demonstrating a significant difference in array characteristics between proximal and distal regions of the cerci, and we also come to a very different conclusion about the re-identifiability of individual mechanoreceptors than did earlier studies.

### Specialized characteristics of the “cercal fovea”

Figures 8, 9, 10 and 12 show that several aspects of the filiform array differ as a function of the distance out along the cerci from the base. First, as recognized in earlier studies and also demonstrated in other crickets species [18], the density of receptors is much greater near the base of the cerci than in regions farther out along the cerci. Second, there are also significantly more transverse hairs in the basal region. This second aspect, in turn, yields different overall directional selectivity curves for the basal and distal regions, as shown in Figure 12.

The 1.5 mm region at the base of the cercus has been referred to as the “cercal fovea” [18], [33], and is the region that also contains the entire array of clavate mechanoreceptors [2], [34]–[37]. The clavate sensors mediate sensitivity to gravity and acceleration, and are all localized to a patch on the baso-medial face of the cerci, within which there are very few filiform hairs. We did not record the anatomical data for the clavate hairs, nor for any of the filiform hairs within these clavate patches, which accounts for the “scooped-out” regions in the vector fields near the base of the cerci in Figures 8, 10 and 13. The presence of this patch of clavate hairs on the medial surface is partially responsible for the decrease in relative number of longitudinal hairs in the cercal fovea: farther out along each cercus, the medial surface is populated by longitudinal hairs. The other aspect of the filiform array that contributes to the high ratio of transverse hairs is the extremely high density of hairs in this basal region relative to the density of hairs in more distal regions of the cerci: since the overall density is higher here, and since most of these dense hairs are transverse, then the ratio of these transverse hairs is exaggerated.

This structural organization is understandable from at least two engineering perspectives. First, the cricket's body would be expected to interfere with, re-direct or even obstruct longitudinal air flow in these baso-medial regions, and development of longitudinal hairs in this region might represent a waste of resources. In contrast, a sensitive acceleration sensor would benefit from placement in a region that is shielded from air currents. Hence, this is an effective functional location for the clavate array. Second, as shown in another recent study, the larger basal diameter of the cricket's cercus makes transverse hairs more sensitive to air currents in this region, due to a relative increase in air current velocity around the larger diameter cercal base [18].

The differential directional selectivities of the basal and more distal regions of the cercal array may be of considerable functional significance, considering recent results demonstrating that the cerci function as delay lines [38]. In that study, all filiform sensory afferent axons were shown to have the same propagation speeds to within a small variance, resulting in a significant and systematic differential propagation time for spikes from filiform receptors at different locations along the structure. The delay-line structure was shown to support computations in many of the projecting cercal interneurons (INs) that yield substantial differential sensitivity to the direction and velocity of small-scale naturalistic stimuli sweeping over the cerci. The observed differential directional selectivity plots shown in Figure 12 would suggest that the cercal mechanosensor arrays themselves might be “tuned” to traveling stimuli with complex dynamical and directional characteristics; e.g., a traveling vortex that would stimulate distal portions of the cerci with longitudinal currents, followed by a transverse stimulus to the basal regions. This is consistent with results from an earlier study that demonstrated differential sensitivity of some INs to stimulation of transverse hairs in proximal vs. distal regions of the cercus [33].

### Filiform mechanosensors are not re-identifiable

There is a long history of studies of re-identifiable sensory hairs in insects and arthropods in general. A fascinating, insightful, beautifully illustrated review of foundational work carried out in Drosophila, that is still very relevant today, was published by Curt Stern in 1954[39]. He demonstrated that there were bristles with particular sets of structural and biomechanical properties that could be found at the same relative locations on every normal specimen of a certain strain, and went on to consider the genetic and developmental mechanisms underlying the positioning of the bristles. Since that time, re-identifiable sensory hairs have been documented and studied in a large range of insect species. In fact, Murphey and colleagues demonstrated that cercal clavate hairs (and filiform hairs within the clavate array) in *Acheta domesticus* are uniquely identifiable based on position and birthday [35]–[37]. It has long been hypothesized that the filiform hairs on the cricket cerci outside of the clavate array are also re-identifiable; *i.e.*, that a filiform hair with a particular set of structural and biomechanical properties can be found at approximately the same relative location on every normal cercus [1], [3], [4].

Considering the inter-animal variation in characteristics of the distributions of filiform receptor sockets shown between the sample of three cerci presented here, this certainly cannot be the case. First and foremost, the numbers of receptors varied substantially between the three different specimens, far beyond the level that could be postulated as the worst-case experimental recording error. Even accounting for the possibility of an unrecognized systematic under-count of sockets that were destroyed along the “cut lines” in the filet procedure, we estimate the largest reasonable error to be in the range of five missed sockets per specimen. And as discussed in the Methods section, we took special care to select specimens that had not had any damage to either cercus during earlier instars. As we noted in the Results section associated with Figure 9, the three specimens came from two different batches, and the two specimens with similar characteristics came from the same batch of crickets. However, even the two specimens from the same batch yielded significantly different data. The large variations in total hair numbers, as well as the many differences documented in the figures we present, argue against the re-identifiability of filiform receptors.

In retrospect, it is understandable that the anatomical measurement procedures used by researchers in earlier studies could have lead to the conclusion the filiform hairs were re-identifiable. The strongest claim of the re-identifiability of filiform hairs was based on a study in which examples of the same (putative) identifiable filiform hair was identified in multiple crickets, based on the position, length and directional movement plane of the hair [4]. It was reported that the standard deviations in the mean preferred direction and circumferential location of a given hair were less than 2% of the total parameter range. However, we note that the filiform hairs are not re-identifiable in the strict sense, the density of hairs is very high and the inter-animal variance of several features is very low. To invert the approach used in that earlier study, if an arc-segment corresponding to 2% of the total circumference of a cercus (i.e., a 7° arc) and 5% of the total cercal length (5 mm) is drawn onto equivalent (arbitrary) locations on each of the three cerci specimens shown in Figure 8, then an observer would almost certainly be able to find hairs with essentially identical lengths and directional movement planes, even though those hairs are not necessarily homologous in the strict sense of re-identifiability. A reasonable analogy is a human fingerprint: a 1 mm square test patch projected onto analogous locations on the fingertips of any sample of subjects would very likely contain a ridge/fold cycle, even though the overall patterns would be unique.

Although our results argue against strict re-identifiability of the filiform hairs, the many highly-constrained aspects of the global organization of the array that were seen to be highly conserved between the different samples indicate that the distribution patter of the hairs is not simply random. In fact, there appears to be a high degree of apparent similarity across all specimens in the locations and anatomical characteristics of the longest hairs (i.e., largest diameter sockets) near the base of the cerci. These sockets correspond to the hairs with the earliest birthdays, as is the case for the clavate sensors [35]–[37]. It could very well be the case that the same developmental mechanisms are operating to determine the location of filiform hairs out along the cercus as are operating to determine the location of the identifiable clavate hairs, but that the much larger cercal surface area covered by the filiform hairs enables the inevitable “noise” in the developmental processes to manifest as a higher accumulated level of variation in the locations and even the numbers of hairs inserted into the system during each ecdysial stage.

We note that the large variation in total hairs per specimen could account for the large range of published estimates of the number of hairs/cercus, ranging from 752 hairs/cercus [1] up to 1000–2000/cercus [40]. We note that our specimens were limited to the proximal halves of the three cerci, and that the density of hairs are known to be lower on the distal halves. Thus, a reasonable estimate for the total number of hairs for entire intact cerci would be in the range of between 500–750, more in line with the count by Palka et al. [1].

### Functional significance of non-uniform directional selectivities and receptor densities

It is a general principle that the resolution with which a sensory system can encode information about a particular stimulus parameter depends on the functional packing density of the receptor cells and the degree of overlap of their receptive fields. It is generally assumed to be the case that, all other factors being equal, higher receptor packing density enables higher resolution. Considering the remarkable non-uniformities in the directional selectivities of the sensors in the cercal array, it might be expected that the crickets would have higher directional acuity in the directions corresponding to the peaks in the polar plots shown in Figures 11 **,** 12 and 13. However, no significant non-uniformity in directional selectivity has been found in information theoretic analysis of the set of four interneurons (identified as left- and right-10-2 plus left- and right-10-3) that are known to encode and transmit information about air current stimulus direction to higher centers [41]–[43]. This absence of evidence for non-uniform sensitivity at the output of the cercal system is understandable, however, considering the parametric breadth of the tuning curves for the filiform mechanoreceptors. The directional response curves of the cercal hair receptors are essentially sinusoidal with respect to the stimulus direction angle around the animal, yielding an excitatory response over a full 180 degree range of directions [9]. From a theoretical perspective, there is enough overlap between the tuning curves of receptors in the different peaks of Figure 11D that the non-uniformities are effectively “smoothed out” from a functional standpoint (e.g. see [44]).

A more likely functional correlate of the over-representation of directional sensitivity as four bands on each cercus may also relate to the delay line characteristics of the cerci [38]. Within that context, the concentration of hair movement axes along a restricted subset of directions is understandable within the functional context of signal averaging. Specifically, if it is beneficial to the animal to be able to extract accurate information about the speed with which a low amplitude air front is sweeping along a cercus in a noisy environment, then a linearly distributed arrangement of sensors enabling efficient and effective signal averaging across otherwise identical units would offer a selective advantage.

Even though the individual receptor hairs cannot be considered to be re-identifiable, the inter-animal variation of several global organizational features is low, consistent with constraints imposed by functional effectiveness, energetics, developmental processes and/or developmental legacy. However, it is extremely difficult to assess the relative weighting of these different constraining factors. We suggest that the data presented here could provide a basis for a direct test of the extent to which the packing density of filiform hairs approaches an optimal configuration with respect to function constraints imposed by the fluid dynamics of air flow over this sensory organ. Studies of the biomechanical properties of the filiform receptor hairs in this and similar species indicate that the density of the hairs is high enough that they might interact with one another through their fluid dynamical environment [17], [18], [21]. In particular, the stimulus threshold for any individual hair is likely to depend upon its proximity to other surrounding hairs, and to the movement axes of those hairs. Thus, the highly-conserved global features of the filiform hair array may be of considerable functional significance, and may be the basis for a substantially increased sensitivity of the system over other possible arrangements with different global organization patterns. The fact that some of these characteristics are highly preserved across animals, and that they are important determinants of the cercal stimulus sensitivity, suggest that they would be susceptible to selective pressure and subject to control during development.

## Supporting Information

Spreadsheet S1 **Spreadsheet containing all anatomical measurements used for** **Table 1** **and** **Figures 8** **,** **9** **,** **10** **,** **11** **,** **12** **, and** **13** **.** A separate sheet is provided for each of the three cercus preparations. Definitions of the column headings are provided in a fourth sheet.
(XLSX)
